# A novel formula used for predicting hepatocellular carcinoma after the achievement of sustained virologic response by direct-acting antivirals in patients with chronic hepatitis C

**DOI:** 10.1371/journal.pone.0292019

**Published:** 2023-09-21

**Authors:** Yuji Yoshida, Masanori Atsukawa, Chisa Kondo, Michika Kitamura, Kaori Shioda-Koyano, Tadamichi Kawano, Hiroki Ono, Korenobu Hayama, Tomomi Okubo, Taeang Arai, Norio Itokawa, Katsuhiko Iwakiri

**Affiliations:** 1 Division of Gastroenterology, Nippon Medical School Chiba Hokusoh Hospital, Inzai, Japan; 2 Division of Gastroenterology and Hepatology, Nippon Medical School, Tokyo, Japan; Kaohsiung Medical University, TAIWAN

## Abstract

Although eliminating HCV can prevent hepatocellular carcinoma (HCC), some patients develop HCC even after obtaining sustained virologic response (SVR). Previously, we developed a new formula to predict advanced liver fibrosis. This study aimed to clarify the usefulness of this formula for predicting HCC after achieving SVR. Among 351 consecutive patients who had been treated with direct-acting antivirals, 299 were included in this study. New formula scores were used as a marker for predicting liver fibrosis and as a predictive model for HCC incidence. The participants were 172 men and 127 women with a median age of 68 years. The median new formula score was -1.291. The cumulative HCC incidence rates were 4.3%, 9.7%, and 12.5% at 1, 3, and 5 years, respectively. The cumulative incidence of HCC was significantly higher in patients with a history of HCC than in those without treatment history of HCC (P = 2.52×10^−26^). Multivariate analysis revealed that male (HR = 6.584, 95% CI = 1.291–33.573, P = 0.023) and new formula score (HR = 1.741, 95% CI = 1.041–2.911, P = 0.035) were independent factors associated with the development of HCC in patients without a treatment history of HCC. The optimal cutoff value for predicting the development of HCC was -0.214. The cumulative incidence rates of HCC in patients with new formula scores ≥-0.214 were 5.4%, 15.3%, and 15.3% at 1, 3, and 5 years, respectively, whereas the incidence rates of HCC in patients with new formula scores <-0.214 were 0.0%, 0.6%, and 4.8%, respectively (P = 2.12×10^−4^). In conclusion, this study demonstrated the usefulness of new formula scores as a predictor of HCC after achieving SVR, especially in patients without past treatment history of treatment for HCC.

## Introduction

Recently, interferon-free therapies combined with direct-acting antivirals (DAAs) have been approved worldwide for patients with chronic hepatitis C [1–3]. DAAs have replaced interferon-based therapy as the mainstay treatment for chronic hepatitis C, enabling shorter treatment times, improved safety, and dramatically improved sustained virological response (SVR) rates regardless of the patient’s condition, such as advanced age, advanced liver fibrosis, decompensated cirrhosis, chronic kidney disease, or other complications [4–9]. While chronic hepatitis C can cause hepatocellular carcinoma (HCC) if left untreated, elimination of HCV with antiviral drugs can prevent the development of HCC by DAA treatment as well as by interferon-based treatment [10–15].

However, some patients can develop HCC even after achieving SVR, and as the number of patients with SVR increases dramatically, screening methods for the development of HCC in these patients with SVR become more important. Moreover, patients treated with DAAs are more likely to be elderly and have more advanced liver fibrosis than are those treated with interferon-based therapy [4–9, 16], which naturally leads to a higher risk of liver carcinogenesis after achieving SVR. HCC recurrence may occur among patients who have achieved SVR with DAAs after radical therapy [17–20]. Therefore, a cost-effective and efficient follow-up method is required for patients who achieve SVR is being sought. It remains controversial as to which patients should be actively monitored for hepatocarcinogenesis after achieving SVR.

Many reports have described the characteristics of patients prone to liver carcinogenesis after achieving SVR. As with many reports, factors reflecting the degree of liver fibrosis, such as the presence of liver cirrhosis, high M2BPGi, low platelets, and high FIB-4 index, have a significant impact on the development of HCC after the achievement of SVR [10–15, 17–20].

Previously, we noninvasively combined three factors—hyaluronic acid, type IV collagen 7s, and M2BPGi—to create a new formula for predicting advanced liver fibrosis [21]. This model was superior to the aspartate aminotransferase-to-platelet ratio index (APRI) and the FIB-4 index in its ability to predict the degree of liver fibrosis. Therefore, this study aimed to clarify the usefulness of this new formula for predicting liver carcinogenesis after achieving SVR in patients with chronic hepatitis C.

## Material and methods

### Participants

This retrospective, single-center study investigated the incidence of HCCs in patients who had been treated with DAA. Among 351 consecutive patients who had visited Nippon Medical School Chiba Hokusoh Hospital between March 2014 and November 2020, 299 were included in this study. Data was collected from November 2022 to March 2023 in five-month periods, and the data collected was analyzed in April of 2023.

Among the 351 patients, 52 were excluded for the following reasons: (1) relapse after DAAs treatment (n = 4), (2) inability to assess the therapeutic effect due to missing data (n = 41), and (3) loss to follow-up (n = 7). Liver cirrhosis was diagnosed through imaging (abdominal computed tomography and/or ultrasonography) or liver biopsy. Abdominal ultrasonography, contrast-enhanced computed tomography (CE-CT), and/or CE-MRI were performed as screening tests for HCC. If HCC was suspected by abdominal ultrasonography, CE-CT and/or CE-MRI was then performed to confirm the diagnosis. Patients with preexisting HCC were treated with curative therapy for HCC, whereas those without HCC on imaging were treated with DAAs. After DAAs therapy, all patients underwent imaging studies, such as abdominal ultrasonography, abdominal CE-CT, or MRI, at least twice a year. This study conformed with the ethical guidelines of the Declaration of Helsinki and approved by the institutional review board of each participating institution (approval number 675–2 of Nippon Medical School Chiba Hokusoh Hospital). Written informed consent was obtained from each patient before enrollment in the study.

### Liver fibrosis markers

Type IV collagen 7S, hyaluronic acid, and Wisteria floribunda agglutinin-positive Mac-2 binding protein (WFA+-M2BP) levels were measured as markers of liver fibrosis to predict HCC development. As we had previously reported, new formula scores = -6.154 + 1.166 × ln type IV collagen 7S + 0.526 × ln hyaluronic acid + 1.069 × WFA+-M2BP, which is a combination of the above three factors [21], was used as a marker for predicting liver fibrosis as a predictive model for incidence of HCCs. The APRI was calculated as follows: {[AST (U/L) / AST upper limit of normal (U/L)] / platelet count (×10^9^/L)} × 100. The FIB-4 index was calculated as follows: [age (years) × AST (U/L)] / [platelet count (×10^9^/L) × ALT (U/L) ^1/2^]. The Albumin-Bilirubin (ALBI) score was calculated based on the serum albumin and total bilirubin values using the following formula: ALBI score = [log10 bilirubin (mol/L)×0.66] + [albumin (g/L)×−0.085].

### Statistical analyses

Categorical data were expressed as numbers. Continuous data were expressed as medians and ranges. Cox proportional hazards regression analyses were used to identify factors associated with the development of HCC within 5 years of achieving SVR. Cumulative HCC incidence rates were generated using the Kaplan–Meier method and compared using the log-rank test. A receiver operating characteristic (ROC) curve was used to determine the optimal cutoff value of the new formula score for predicting HCC. Statistical significance was set at p < 0.05. significant. All statistical analyses were performed using IBM SPSS Statistics (version 26.0; IBM, Tokyo, Japan).

## Results

### Patient characteristics

The participants included 172 male and 127 female. The median patient age was 68 years (range: 27–90 years). The median observation period was 29 months (range: 1.0–72.0 months) after the achievement of SVR. The DAA treatment regimens were daclatasvir/asunaprevir in 41 patients, elbasvir/grazoprevir in 36, glecaprevir/pibrentasvir in 63, sofosbuvir/ledipasvir in 47, ombitasvir/paritaprevir/ritonavir ± ribavirin in 65, and sofosbuvir/ribavirin in 47 patients. The median platelet count before the start of DAA treatment was 154×10^3^/μL (47–496×10^3^/μL), FIB-4 Index <3.25 in 171 and ≥3.25 in 128 patients. Median Type IV collagen 7S, Hyaluronic acid and WFA+M2BP were 4.9 ng/mL (1.9–17.8 ng/mL), 88.8 ng/mL (1.5–1812.2 ng/mL) and 2.01 C.O.I. (0.22–17.44 C.O.I.), respectively. The median new formula score was −1.291 (−5.490–3.165). Twenty-five patients with a history of pre-treatment HCC and 274 with no history of treatment were included. Table 1 also shows the background by the presence or absence of HCC after achievement of SVR after DAA treatment. There were no patients in this cohort with HBV/HCV co-infection.

**Table 1:** Baseline characteristics of the patients.

Variable	Total N = 299	Patients without new HCCs presence after achievement of SVR N = 274	Patients with new HCCs presence after achievement of SVR N = 25
Sex (Men/ Women)	172/ 127	154/ 120	18/ 7
Age (years)	68 (27–90)	68 (27–90)	78 (47–90)
Platelet count (×10^3^/μL)	154 (47–496)	159 (47–496)	116 (59–246)
AST (U/L)	42 (11–294)	40 (11–294)	61 (22–155)
ALT (U/L)	38 (9–371)	38 (9–371)	48 (12–105)
*γ*GTP (U/L)	36 (6–590)	35 (6–479)	60 (16–590)
Serum albumin (g/dL)	4.0 (2.6–4.6)	4.0 (3.0–4.6)	3.8 (2.6–4.5)
Total Bilirubin (mg/dL)	0.6 (0.2–2.0)	0.6 (0.2–2.0)	0.7 (0.2–1.8)
Type IV collagen 7S (ng/mL)	4.9 (1.9–17.8)	4.8 (1.9–17.8)	6.9 (3.8–12.4)
Hyaluronic acid (ng/mL)	88.8 (1.5–1812.2)	81.7 (1.5–1812.2)	230.3 (44.9–1337.8)
WFA^+^-M2BP (C.O.I.)	2.01 (0.22–17.44)	1.89 (0.22–17.44)	4.51 (1.09–15.61)
AFP (ng/mL)	3.9 (0.7–494.2)	3.7 (0.7–494.2)	8.4 (1.6–98.8)
APRI	0.889 (0.218–9.570)	0.860 (0.218–9.570)	2.241 (0.530–6.379)
FIB-4 index	2.95 (0.50–115.78)	2.83 (0.50–20.93)	7.42 (2.21–115.78)
ALBI score	-2.74 (-3.36- -1.50)	-2.76 (-3.36- -1.55)	-2.54 (-3.08- -1.50)
New formula score	-1.291 (-5.490–3.165)	-1.386 (-5.490–3.165)	0.734 (-2.304–2.605)
Liver cirrhosis (absence/presence)	240 / 59	229 / 45	11 / 14
Diabetes mellitus (absence/presence)	238 / 61	221 / 53	17 / 8

Categoric values are given as number. Continuous variables are given as median (range).

AST, aspartate aminotransferase; ALT, alanine aminotransferase; γ GTP, γ- glutmyltransferase; WFA^+^-M2BP, Wisteria floribunda agglutinin positive Mac-2-binding protein; C.O.I., cutoff index; AFP, alpha fetoprotein; APRI, aspartate aminotransferase to platelet ratio index; ALBI score, albumin-bilirubin score; HCC, hepatocellular carcinoma.

### Cumulative incidence of HCC in all patients after the achievement of SVR

**Fig 1:**
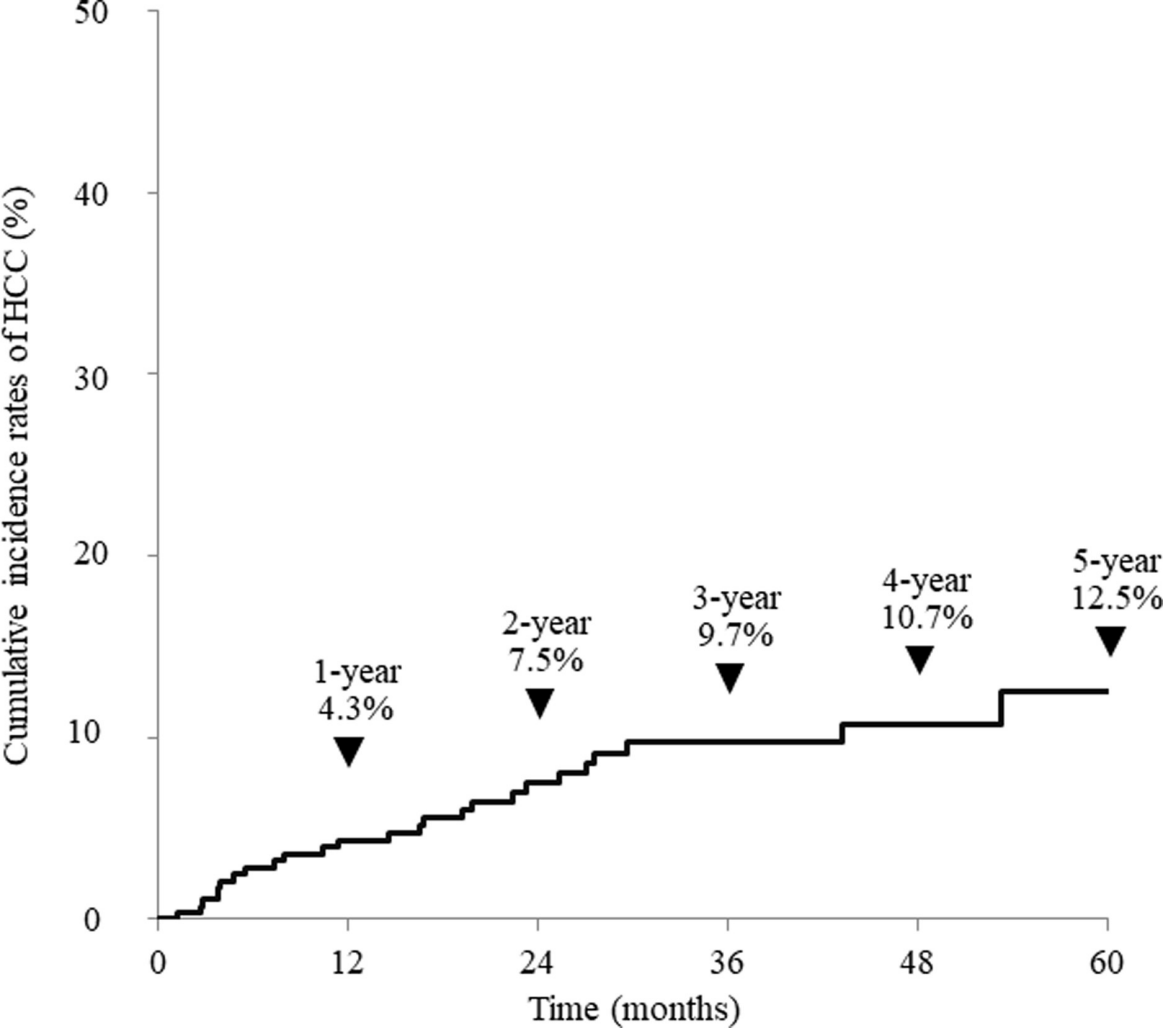
Cumulative incidence of hepatocellular carcinoma in patients who had achieved sustained virologic response.

**Fig 2:**
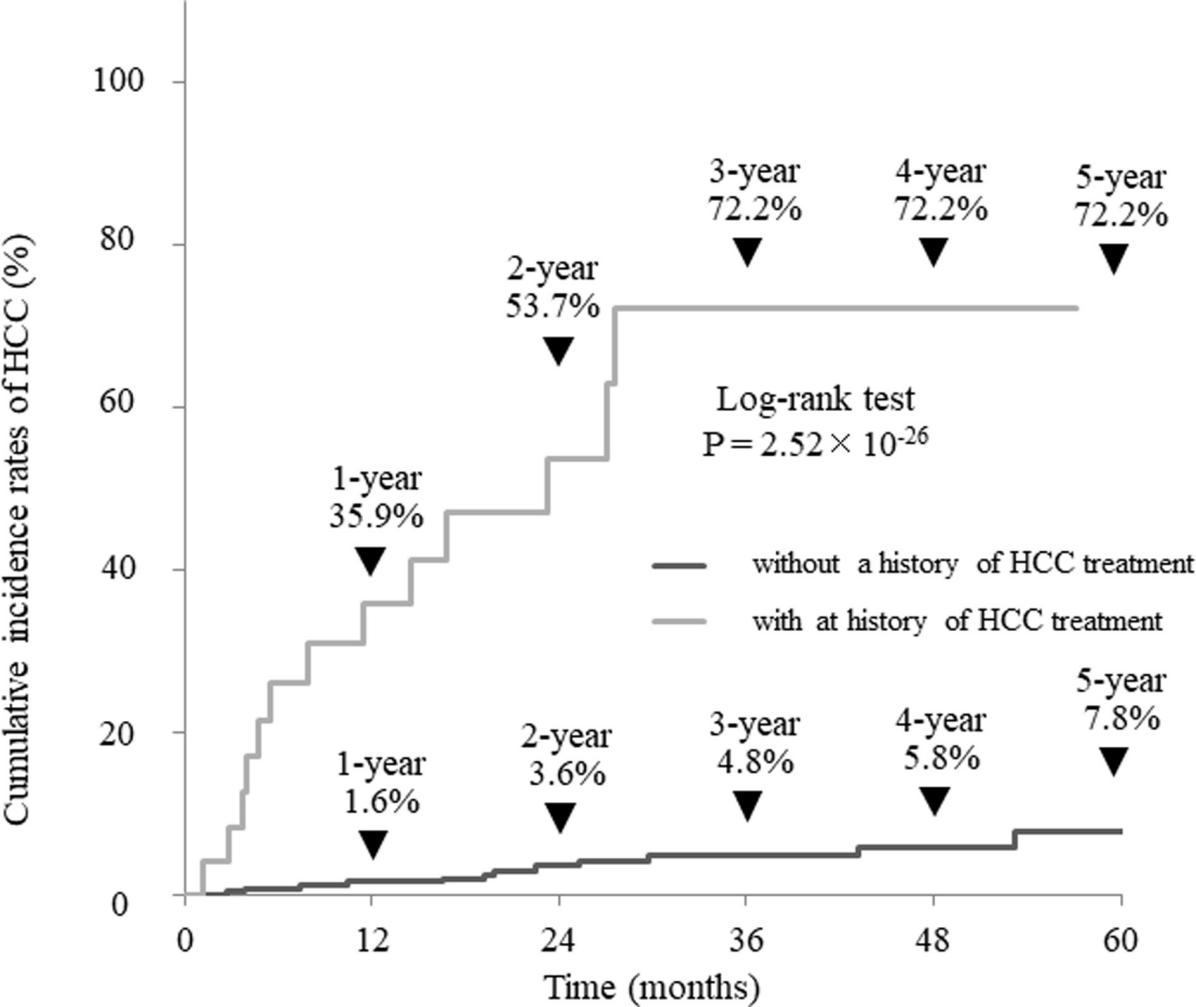
Cumulative incidence of hepatocellular carcinoma with or without a treatment history of hepatocellular carcinoma.

Cumulative HCC incidence was 4.3% at 1 year, 9.7% at 3 years, and 12.5% at 5 years after the achievement of SVR (Fig 1). The cumulative incidence of HCC was significantly higher in the patients with a history of HCC (35.9%, 72.2%, and 72.2% at one, three, and five years) compared with 1.6%, 4.8%, and 7.8% in, patients without treatment history of HCC. The proportion was significantly higher in patients with a history of HCC treatment (P = 2.52×10^−26^, Fig 2).

### Factors associated with HCC after the achievement of SVR

In the univariate analysis of the factors associated with HCC after SVR, age, past treatment history of HCC, γ GTP, liver cirrhosis, new formula score, APRI, ALBI score, and FIB-4 index were identified as significant factors in all 299 patients. Multivariate analysis revealed that past treatment history of HCC (hazard ratio [HR] = 14.527, 95% confidence interval [CI] = 5.560–37.951, P = 4.72×10^−8^), γ GTP (HR = 1.005, 95% CI = 1.001–1.008, P = 0.010), new formula score (HR = 1.389, 95% CI = 1.001–1.925, P = 0.049), and ALBI score (HR = 3.102, 95% CI = 1.055–9.125, P = 0.040) were identified as independent factors associated with the development of HCC after DAA treatment (Table 2).

**Table 2:** Univariate and multivariate analyses using Cox proportional hazards regression of baseline factors associated with development of hepatocellular carcinoma among the 299 patients.

		Univariate			Multivariate		
Variables	Category	HR	95% CI	P value	HR	95%CI	P value
Age (years)	per 1 year up	1.103	1.049–1.159	1.28×10^−4^	1.040	0.971–1.113	0.265
Gender	Male	2.258	0.942–5.412	0.068			
Past treatment history of HCC	yes	21.711	9.719–48.499	6.11×10^−14^	14.527	5.560–37.951	4.72×10^−8^
Diabetes	yes	1.954	0.843–4.531	0.118			
*γ*GTP (U/L)	per 1.0 U/L up	1.005	1.002–1.008	8.93×10^−4^	1.005	1.001–1.008	0.010
AFP	per 1.0ng/mL up	1.002	0.995–1.009	0.561			
Liver cirrhosis	yes	6.734	3.032–14.955	2.80×10^−6^	1.603	0.508–5.060	0.421
New formula score	per 1.0 up	1.818	1.419–2.328	2.27×10^−6^	1.389	1.001–1.925	0.049
APRI	per 1.000 up	1.384	1.048–1.828	0.022	0.444	0.069–2.870	0.394
ALBI score	per 1.00 up	5.112	2.002–13.055	6.48×10^−4^	3.102	1.055–9.125	0.040
FIB4 index	per 1.00 up	1.064	1.016–1.114	8.15×10^−3^	1.202	0.838–1.722	0.317

HCC, hepatocellular carcinoma; γ GTP, γ- glutmyltransferase; AFP, alpha fetoprotein; APRI, aspartate aminotransferase to platelet ratio index; ALBI score, albumin-bilirubin score; HR, hazard ratio; CI, confidence interval

Next, we analyzed factors associated with the development of HCC in 274 patients without a history of HCC treatment. In the univariate analysis, age, male gender, liver cirrhosis, new formula score, APRI, and ALBI score were identified as significant factors. Multivariate analysis revealed that male (HR = 6.584, 95% CI = 1.291–33.573, P = 0.023) and new formula score (HR = 1.741, 95% CI = 1.041–2.911, P = 0.035) were identified as independent factors associated with development of HCC (Table 3). To compare the performance of these factors such as FIB-4 index, new formula score and liver cirrhosis on predicting HCC development and avoid collinearity we built three models of uni-multivariate analyses (model 1 includes FIB-4, model 2 includes new formula, and model 3 includes presence of liver cirrhosis) (S1 Table). For univariate analysis, age, sex, diabetes status, γGTP, and AFP were entered, and FIB-4 index, new formula score, and liver cirrhosis were added to each of the three models. As a result, the new formula score had the lowest p value (p = 9.40×10^−4^), suggesting that it may have better diagnostic performance than the FIB-4 index (p = 1.99×10^−3^) or the presence of cirrhosis (p = 0.015).

**Table 3:** Univariate and multivariate analyses using Cox proportional hazards regression of baseline factors associated with development of HCC in the 274 patients without past treatment of HCC.

		Univariate			Multivariate		
Variables	Category	HR	95% CI	P value	HR	95%CI	P value
Age (years)	per 1 year up	1.096	1.020–1.178	0.013	1.046	0.980–1.116	0.176
Gender	Male	4.648	1.018–21.228	0.047	6.584	1.291–33.573	0.023
Diabetes	yes	0.834	0.183–3.812	0.815			
*γ*GTP (U/L)	per 1.0 U/L up	1.004	0.999–1.010	0.150			
AFP	per 1.0ng/mL up	1.004	0.996–1.011	0.357			
Liver cirrhosis	yes	6.572	2.104–20.531	1.19×10^−3^	0.459	0.110–3.455	0.449
New formula score	per 1.0 up	1.950	1.361–2.794	2.76×10^−4^	1.741	1.041–2.911	0.035
APRI	per 1.000 up	1.445	1.018–2.052	0.040	1.312	0.760–2.266	0.330
ALBI score	per 1.00 up	9.575	3.172–28.899	6.12×10^−5^	2.974	0.463–19.102	0.251
FIB4 index	per 1.00 up	1.063	0.995–1.136	0.070			

γ GTP, γ- glutmyltransferase; AFP, alpha fetoprotein; APRI, aspartate aminotransferase to platelet ratio index; ALBI score, albumin-bilirubin score; HR, hazard ratio; CI, confidence interval

The rate of development of HCC after achievement of SVR by first-generation DAA treatment was 7.4% (10/135), while the rate of development of HCC after achievement of SVR by second-generation DAA treatment was 9.1% (15/164), not significantly different between the two groups (P = 0.677).

### Cumulative HCC incidence by gender and new formula score in patients without treatment history of HCC

**Fig 3:**
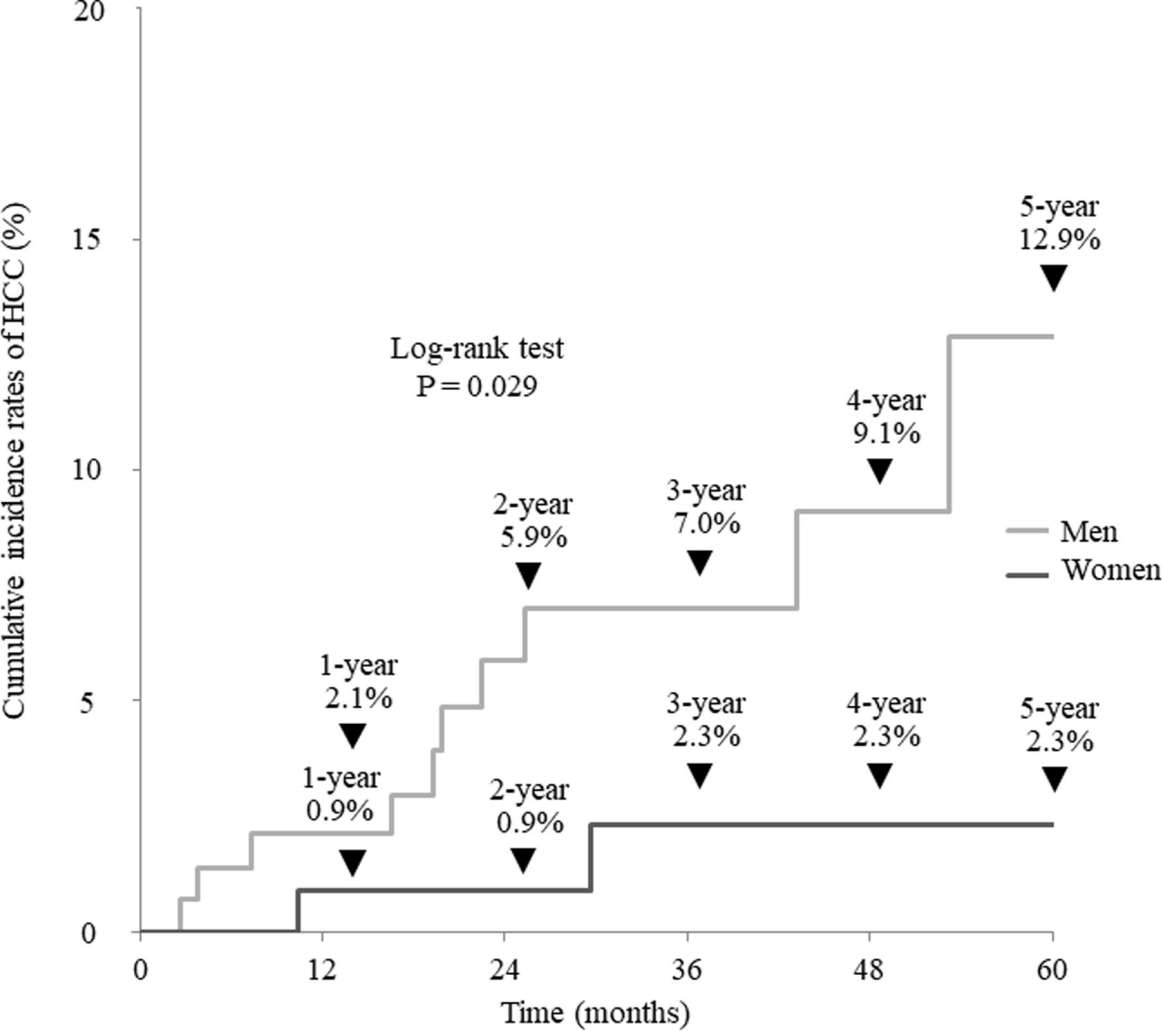
Cumulative incidence of hepatocellular carcinoma without a treatment history of hepatocellular carcinoma according to sex.

**Fig 4:**
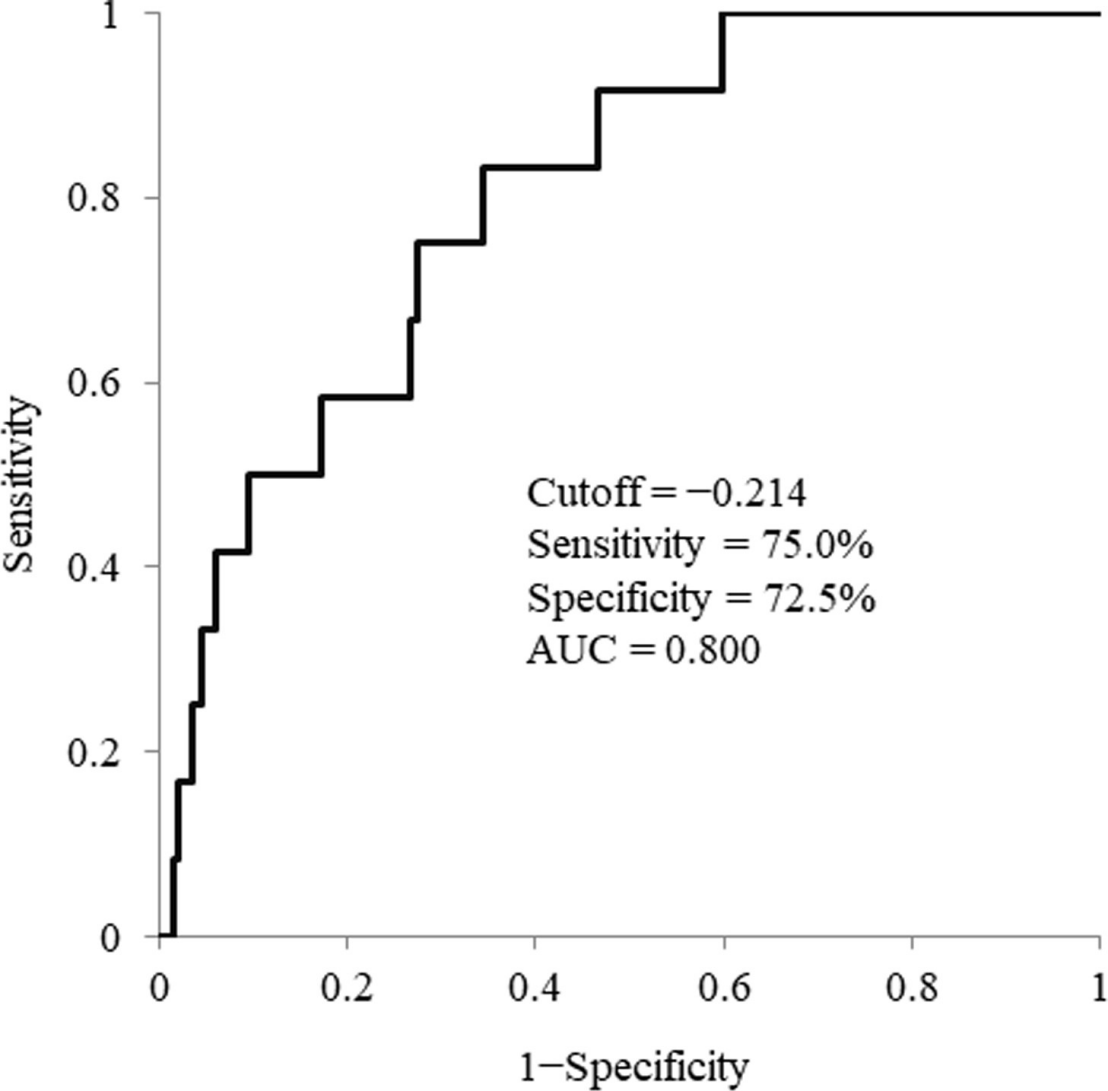
Optimal cutoff values of new formula scores for predicting the development of hepatocellular carcinoma in 274 patients without a treatment history of hepatocellular carcinoma.

**Fig 5:**
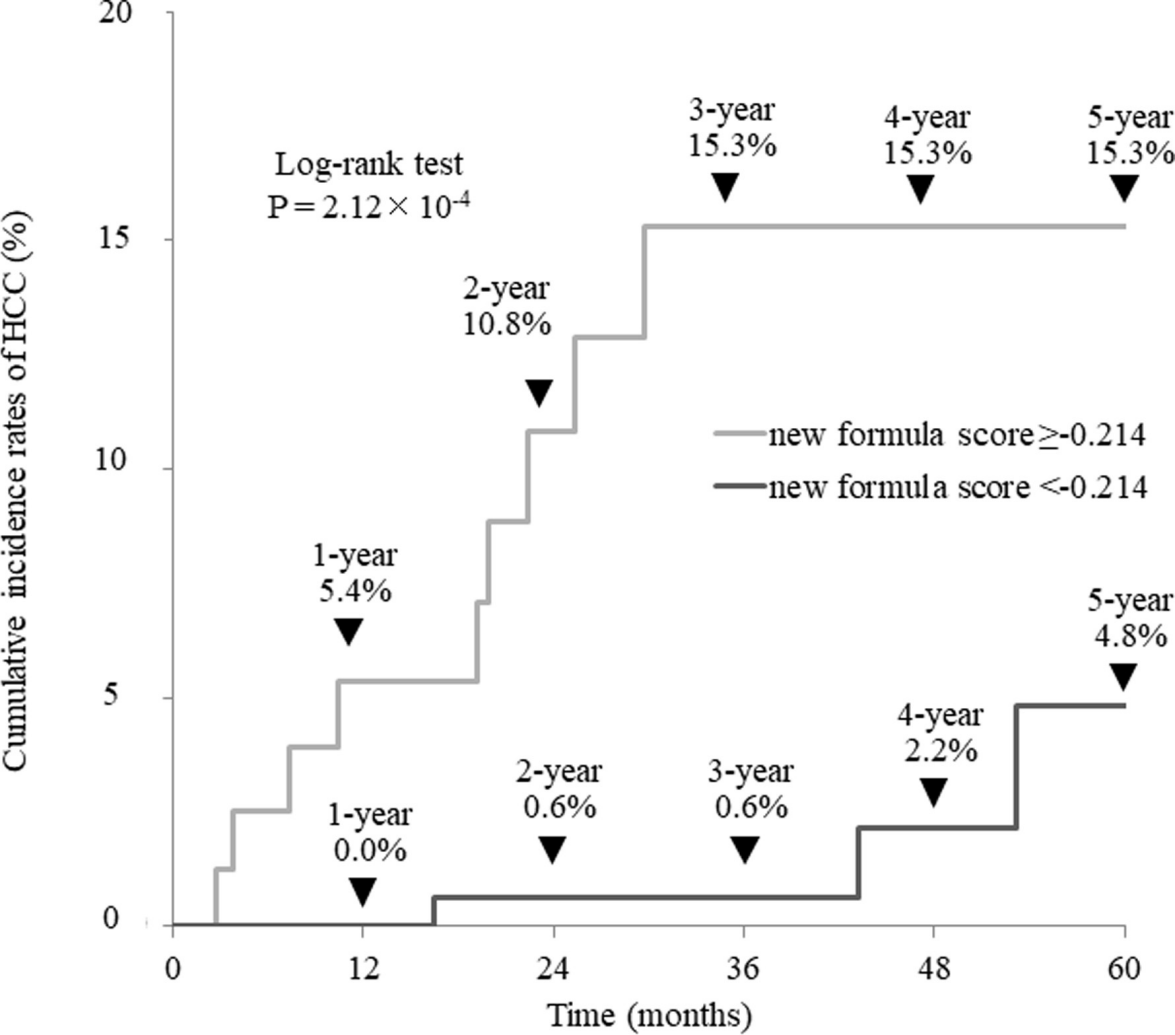
Cumulative incidence of hepatocellular carcinoma without a treatment history of hepatocellular carcinoma according to new formula scores.

The cumulative incidence of HCC after achieving SVR was examined in 274 patients without a history of treatment for HCC. The cumulative HCC incidence rates were 2.1%, 5.9%, 7.0%, 9.1%, and 12.9% for men at 1, 2, 3, 4, and 5 years, respectively, compared with 0.9%, 0.9%, 2.3%, 2.3%, and 2.3% for women, indicating significantly lower rates of HCC (P = 0.029, Fig 3). The optimal cutoff value for predicting the development of HCC in these 274 patients was −0.214 by ROC analysis (sensitivity = 75.0%; specificity = 72.5%; AUC = 0.800, Fig 4). The cumulative incidence rates of HCC in patients with new formula scores ≥−0.214 were 5.4%, 10.8%, 15.3%, 15.3%, and 15.3% at 1, 2, 3, 4, and 5 years, respectively, whereas the incidence rates of HCC in patients with new formula scores <−0.214 were 0.0%, 0.6%, 0.6%, 2.2%, and 4.8%, respectively, indicating a significantly lower rate of HCC (P = 2.12×10^−4^, Fig 5). All patients who developed HCC despite having low new formula scores, were men with a history of alcohol consumption.

## Discussion

Patients with chronic hepatitis C are at a high risk of developing HCC after achieving SVR with DAA therapy [10–15, 17–20]. Kanwal et al. reported that a retrospective cohort study of 18,076 patients showed that 544 patients developed HCC during a mean observation period of 2.9 years, with cumulative HCC incidence rates of 1.1% at 1 year, 1.9% at 2 years, and 2.8% at 3 years. The factor most strongly associated with HCC development was the presence of liver cirrhosis, but the risk of HCC development was higher in patients with a continuously high FIB-4 Index and APRI, regardless of the presence or absence of liver cirrhosis [11]. In a retrospective cohort study of 48,135 patients who achieved SVR with antiviral therapy between 2000 and 2015, with a mean observation period of 5.4 years, Ioannou et al. reported the long-term evolution of the risk of developing HCC in patients who achieved SVR with IFN-free DAA combination therapy [12]. In this report, cirrhosis patients remained at a high risk of developing HCC for a long period, even after improvement in the FIB-4 Index after the achievement of SVR, and that even non-cirrhosis patients with a high FIB-4 Index are at a high risk of developing HCC. Furthermore, Ide et al. conducted a multicenter prospective study of 2552 patients with chronic hepatitis C who achieved SVR with DAAs therapy [13]. The cumulative incidence of HCC was 1.3% at one year, 2.9% at two years, and 4.9% at three years, and the incidence of HCC was significantly higher in cirrhosis patients than in non-cirrhosis patients. Multivariate analysis revealed that older age, male sex, high-GTP level, and high FIB-4 Index were independent factors associated with the development of HCC after achieving SVR. Thus, the degree of liver fibrosis is closely associated with the development of HCC after SVR.

Recently, the measurement of liver stiffness by elastography has been reported as a noninvasive method for predicting liver fibrosis. However, the FibroScan is an expensive device that is difficult to install. Previously, we had developed a new formula to predict advanced liver fibrosis based on FibroScan results [21]. This score was superior to the APRI and FIB-4 index in predicting the degree of liver fibrosis.

Toyoda et al. noted that surveillance for the development of HCC in patients after achieving SVR varies by country and region [22]. In other words, they emphasized that the frequency and methods of imaging and blood tests vary by region. Therefore, the characteristics of HCC development vary across patients who achieve SVR using a local cohort. Therefore, this study analyzed how the new formula, a potential indicator of liver fibrosis that we had previously developed for patients at our institution, is associated with the development of HCC after the achievement of SVR. Treatment history of HCC was the most important factor in the development of HCC after achieving SVR in the entire cohort, similar to previous reports [17–20]. However, the new formula score was extracted as an independent factor along with γ GTP and ALBI score, independent of past treatment history of HCC, indicating that the new formula score is useful in predicting the development of HCC after achievement of SVR. In addition, the new formula score was extracted as an independent and significant factor for the development of HCC after the achievement of SVR, independent of male gender, when limited to patients without a treatment history of HCC. Notably, the APRI and FIB-4 index, which represent the degree of liver fibrosis similar to the new formula score, were not extracted as independent factors in predicting development of HCC in either the overall analysis or the analysis of the patients without past treatment history of HCC. As reported previously [13, 20, 23, 24], the incidence of HCC after the achievement of SVR was clearly more common in men, being 15.3% in men at 5 years, compared to 4.8% in women. Furthermore, the incidence of HCC was 15.3% at 3 years for patients with new formula scores ≥−0.214 or higher, compared with 0.6% at 3 years for patients with new formula scores <−0.214. In addition, a detailed examination of HCC cases among those with new formula scores <−0.214 revealed that all of them were men with a history of alcohol consumption. Moreover, alcohol consumption after achieving SVR is closely associated with subsequent development of HCC [25, 26]. Thus, it in patients with low new formula scores in the absence of lifestyle habits associated with the development of HCC.

A limitation of this study was its single-center study design. Second, the sample size was small. Third, the follow-up period was only 5 years; therefore, further long-term follow-up is required.

In conclusion, this study showed the usefulness of the new formula as a predictor of HCC after DAA treatment, especially in patients without past treatment history of HCC. Other factors associated with the development of HCC included male sex and history of alcohol consumption. Therefore, the results of this study require further validation in a larger cohort.

## Supporting information

S1 Table Univariate and multivariate analyses using Cox proportional hazards regression of baseline factors associated with development of HCC in the 274 patients without past treatment of HCC. (DOCX)

S1 Data (XLSX)
