# Supplementary material for: A novel formula used for predicting hepatocellular carcinoma after the achievement of sustained virologic response by direct-acting antivirals in patients with chronic hepatitis C
Source: PLoS One. 2023 Sep 21;18(9):e0292019. doi: 10.1371/journal.pone.0292019 (PMC10513247; doi:10.1371/journal.pone.0292019)
Supplement: S1 Table — (DOCX) [file pone.0292019.s001.docx]

**Suppl. Table 1 Univariate and multivariate analyses using Cox proportional hazards regression of baseline factors associated with development of HCC in the 274 patients without past treatment of HCC.**

**(model1)**

|  | | Univariate | | | Multivariate | | |
| --- | --- | --- | --- | --- | --- | --- | --- |
| Variables | Category | HR | 95% CI | P value | HR | 95%CI | P value |
| Age (years) | per 1 year up | 1.096 | 1.020-1.178 | 0.013 | 1.086 | 1.015-1.162 | 0.017 |
| Gender | Male | 4.648 | 1.018-21.228 | 0.047 | 8.145 | 1.464-45.318 | 0.017 |
| Diabetes | yes | 0.834 | 0.183-3.812 | 0.815 |  |  |  |
| γGTP (U/L) | per 1.0 U/L up | 1.004 | 0.999-1.010 | 0.150 |  |  |  |
| AFP | per 1.0ng/mL up | 1.004 | 0.996-1.011 | 0.357 |  |  |  |
| FIB-4 index | per 1.00 up | 1.063 | 0.995-1.136 | 0.070 | 1.214 | 1.074-1.373 | 1.99×10^-3^ |

γ GTP, γ- glutmyltransferase; AFP, alpha fetoprotein; APRI, aspartate aminotransferase to platelet ratio index; ALBI score, albumin-bilirubin score; HR, hazard ratio; CI, confidence interval

**(model2)**

|  | | Univariate | | | Multivariate | | |
| --- | --- | --- | --- | --- | --- | --- | --- |
| Variables | Category | HR | 95% CI | P value | HR | 95%CI | P value |
| Age (years) | per 1 year up | 1.096 | 1.020-1.178 | 0.013 | 1.061 | 0.997-1.128 | 0.061 |
| Gender | Male | 4.648 | 1.018-21.228 | 0.047 | 6.150 | 1.321-28.643 | 0.021 |
| Diabetes | yes | 0.834 | 0.183-3.812 | 0.815 |  |  |  |
| γGTP (U/L) | per 1.0 U/L up | 1.004 | 0.999-1.010 | 0.150 |  |  |  |
| AFP | per 1.0ng/mL up | 1.004 | 0.996-1.011 | 0.357 |  |  |  |
| New formula score | per 1.0 up | 1.950 | 1.361-2.794 | 2.76×10^-4^ | 1.953 | 1.314-2.904 | 9.40×10^-4^ |

γ GTP, γ- glutmyltransferase; AFP, alpha fetoprotein; HR, hazard ratio; CI, confidence interval

**(model3)**

|  | | Univariate | | | Multivariate | | |
| --- | --- | --- | --- | --- | --- | --- | --- |
| Variables | Category | HR | 95% CI | P value | HR | 95%CI | P value |
| Age (years) | per 1 year up | 1.096 | 1.020-1.178 | 0.013 | 1.074 | 1.007-1.146 | 0.030 |
| Gender | Male | 4.648 | 1.018-21.228 | 0.047 | 4.315 | 0.936-19.898 | 0.061 |
| Diabetes | yes | 0.834 | 0.183-3.812 | 0.815 |  |  |  |
| γGTP (U/L) | per 1.0 U/L up | 1.004 | 0.999-1.010 | 0.150 |  |  |  |
| AFP | per 1.0ng/mL up | 1.004 | 0.996-1.011 | 0.357 |  |  |  |
| Liver cirrhosis | yes | 6.572 | 2.104-20.531 | 1.19×10^-3^ | 4.325 | 1.334-14.025 | 0.015 |

γ GTP, γ- glutmyltransferase; AFP, alpha fetoprotein; HR, hazard ratio; CI, confidence interval
